# Fatal Cardiac Arrhythmia and Long-QT Syndrome in a New Form of Congenital Generalized Lipodystrophy with Muscle Rippling (CGL4) Due to *PTRF-CAVIN* Mutations

**DOI:** 10.1371/journal.pgen.1000874

**Published:** 2010-03-12

**Authors:** Anna Rajab, Volker Straub, Liza J. McCann, Dominik Seelow, Raymonda Varon, Rita Barresi, Anne Schulze, Barbara Lucke, Susanne Lützkendorf, Mohsen Karbasiyan, Sebastian Bachmann, Simone Spuler, Markus Schuelke

**Affiliations:** 1 Genetics Unit, Ministry of Health, Directorate General of Health Affairs, Royal Hospital, Muscat, Oman; 2 Institute of Human Genetics, International Center for Life, Newcastle University, Newcastle upon Tyne, United Kingdom; 3 Department of Rheumatology, Alder Hey Children's Hospital, Liverpool, United Kingdom; 4 Department of Neuropediatrics, Charité University Medical School, Berlin, Germany; 5 NeuroCure Clinical Research Center, Charité University Medical School, Berlin, Germany; 6 Institute of Human Genetics, Charité University Medical School, Berlin, Germany; 7 Muscle Research Unit, Experimental and Clinical Research Center, Charité University Medical School, Berlin, Germany; 8 Department of Anatomy, Charité University Medical School, Berlin, Germany; 9 Core Facility for Electron Microscopy, Charité University Medical School, Berlin, Germany; Stanford University School of Medicine, United States of America

## Abstract

We investigated eight families with a novel subtype of congenital generalized lipodystrophy (CGL4) of whom five members had died from sudden cardiac death during their teenage years. ECG studies revealed features of long-QT syndrome, bradycardia, as well as supraventricular and ventricular tachycardias. Further symptoms comprised myopathy with muscle rippling, skeletal as well as smooth-muscle hypertrophy, leading to impaired gastrointestinal motility and hypertrophic pyloric stenosis in some children. Additionally, we found impaired bone formation with osteopenia, osteoporosis, and atlanto-axial instability. Homozygosity mapping located the gene within 2 Mbp on chromosome 17. Prioritization of 74 candidate genes with *GeneDistiller* for high expression in muscle and adipocytes suggested *PTRF-CAVIN* (Polymerase I and transcript release factor/Cavin) as the most probable candidate leading to the detection of homozygous mutations (c.160delG, c.362dupT). PTRF-CAVIN is essential for caveolae biogenesis. These cholesterol-rich plasmalemmal vesicles are involved in signal-transduction and vesicular trafficking and reside primarily on adipocytes, myocytes, and osteoblasts. Absence of PTRF-CAVIN did not influence abundance of its binding partner caveolin-1 and caveolin-3. In patient fibroblasts, however, caveolin-1 failed to localize toward the cell surface and electron microscopy revealed reduction of caveolae to less than 3%. Transfection of full-length PTRF-CAVIN reestablished the presence of caveolae. The loss of caveolae was confirmed by Atomic Force Microscopy (AFM) in combination with fluorescent imaging. PTRF-CAVIN deficiency thus presents the phenotypic spectrum caused by a quintessential lack of functional caveolae.

## Author Summary

Patients with generalized lipodystrophy have a marked lack of body fat. Several gene defects have been described that impede fat synthesis and maturation of fat cells. Here we report on mutations in a novel gene, called *PTRF-CAVIN*, causing congenital generalized lipodystrophy type 4 (CGL4) that is additionally associated with muscle disease. Patients' muscles are large but weak and show an involuntary, rolling contraction pattern called “rippling.” Further symptoms comprise life-threatening cardiac arrhythmias and a disorder of bone formation. We searched for shared segments in the genome of seven patients and found the responsible gene, called *PTRF-CAVIN,* on chromosome 17. This gene is crucial for caveolae (latin for “small caves”) formation. These small indentations of the cell membrane are found on the surface of muscle, bone, fat, and immune cells and facilitate cell-to-cell communication and the absorption of substances from the extracellular space. Patients lack more than 97% of caveolae and artificial insertion of the correct gene into patient skin cells led to the reappearance of caveolae. As cardiac arrhythmia is a severe and potentially life-threatening condition, patients with CGL4 should be closely monitored by ECG and, if necessary, fitted with an implanted pacemaker and cardioverter defibrillator (ICD) device.

## Introduction

Congenital generalized lipodystrophies (CGL1-3, Berardinelli-Seip syndrome, MIM 608594, 269700, 612526) are autosomal recessive disorders characterized by almost complete absence of body fat associated with dyslipidemia and insulin resistance [1],[2]. The discovery of different CGL-related gene defects confirmed its genetic heterogeneity and shed new light on adipocyte function. Causative mutations were found in an adipocyte differentiation factor (Seipin, *BSCL2*) [3], in an enzyme of the triglyceride and glycerophospholipid biosynthetic pathway (*AGPAT2*) [4], and in a lipid-binding protein essential for the formation of caveolae on adipocytes (*CAV1*) [5],[6].

Based on the clinical phenotype and genotyping results of patients from consanguineous Omani families, Rajab *et al.* delineated a novel, genetically distinct CGL-subtype (CGL4) [7],[8]. In contrast to the “classic” variants, symptoms were more widespread comprising myopathy, smooth and skeletal muscle hypertrophy, cardiac arrhythmias, osteopenia and distal metaphyseal deformation with joint stiffness. These peculiarities suggested an associated problem both with myocyte growth/function and bone formation. A recent report on two siblings with generalized lipodystrophy, muscle weakness and cervical instability also reminded us of this phenotype [9].

Here we report on the clinical and genetic evaluation of patients and their family members with CGL4 from Oman (n = 10) and the UK (n = 1) and describe the discovery of mutations in the gene *PTRF-CAVIN* (Polymerase I and transcript release factor/Cavin) which is an essential factor for the biogenesis of caveolae.

## Results

### Case histories

#### Family I

**Figure 1:**
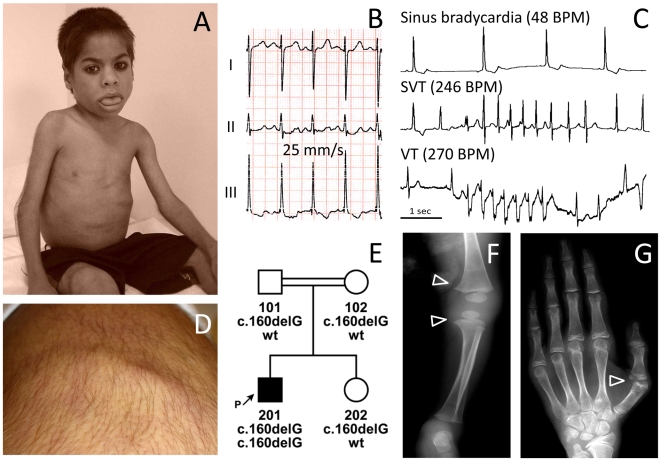
Phenotype of patient FI:201 from Oman. (A) Patient at the age of 4 years with macroglossia, grossly reduced subcutaneous fat tissue and a protruding abdominal wall. (B) ECG of the patient with a QTc (Bazett) of 480 ms. (C) Sections of 24h ECG Holter-monitoring show a complex cardiac arrhythmia with intermittent sinus bradycardia, supraventricular (SVT) and ventricular tachycardia (VT). BPM, beats per minute (D) Percussion-induced, local prolonged contractions (“mounding”) at the quadriceps muscle persisting for 2–3 seconds. (E) Pedigree of the consanguineous family and genotypes of the family members. (F) X-ray radiograph of the knees at 9 months of age, showing broadening of the distal metaphyses (arrowheads) and osteopenia. (G) X-ray radiograph of the left hand at the age of 13.5 years showing osteoporosis and osteopenia. The metacarpo-phalangeal joint of the thumb shows arthritic changes and a partial dislocation with ulnar deviation (arrowhead).

The now 14-year-old boy (FI:201, Figure 1E) was born to first degree cousins from Oman. His sister is healthy. After birth he presented with paucity of subcutaneous fat, increased amount of body hair, a protruding abdomen and a large tongue (Figure 1A). Congenital hypothyroidism was excluded. At 6 weeks a hypertrophic pyloric stenosis was treated by pyloromyotomy. He had poor appetite and failure to thrive, with weight 3SD below the mean. He had episodic hot flushes and *cutis marmorata* and suffered from frequent staphylococcal skin infections and severe pneumonias. At 10 years of age investigation of frequent palpitations and syncopes revealed both ventricular and supraventricular tachycardia and extrasystoles alternating with sinus bradycardia and post-tachycardic pauses of up to 2.2 sec (Figure 1C). A corrected QT-time (QTc with Bazett's correction) between 450–480 ms (Figure 1B) and T-wave abnormalities (alternating in V2,V3 and notched in V4) indicate an intermediate to high probability of long-QT syndrome [10]. Around the same time he had difficulties swallowing and a barium study revealed esophageal dysmotility. Presently, at 14 years of age, he often feels tired, weak, cannot walk for more than one kilometer and complains of back pain and joint stiffness. His mental development is normal, and he is a very good pupil. He has mild splenomegaly, a liver size at the 95^th^ percentile with normal echogenicity on ultrasound but elevated transaminases (AST 120 U/l; N<23 and ALT 76 U/I; N<29). His bone phenotype comprises osteopenia with enlarged epiphyses (Figure 1F), a bone age retarded by 1.5 years, finger contractures with ulnar deviation (Figure 1G), spinal rigidity and a prominent hyperlordosis. Skin fold thickness was measured with a Holtain-T/W-Skinfold-Caliper (Holtain, Crymych, UK) over the triceps (2.5 mm); biceps (2.9 mm), subscapular region (3.6 mm), and iliac crest (2.8 mm) yielding 6.1% whole body fat (<3^rd^ percentile) [11]. Muscular hypertrophy, especially of the thighs, is associated with limb girdle weakness. “Mounding” (Figure 1D) and **P**ercussion **I**nduced **R**apid **C**ontractions (PIRCs) can be elicited from all larger muscles. These events are silent on EMG thus excluding myotonia. Serum investigations revealed elevated creatine kinase levels (1,898 IU/l; N<169), dyslipidemia with *elevated* serum triglycerides (2.29 mmol/l; N 0.57–1.71), pre-β-lipoproteins (VLDL 39%; N<30) and *reduced* HDL-cholesterol (0.47 mmol/l; N>0.96), apolipoprotein A1 (0.72 g/l; N 1.00–1.50) and B (0.6 g/l; N 0.7–1.2). Serum leptin levels were reduced (0.9 µg/l; N 2.4–24.4, reference range for adult individuals with a BMI below the 25^th^ percentile). Insulin resistance was increased with a HOMA-IR index of 3.2 (N 0.97–2.27) without any signs of *acanthosis nigricans*. Fasting glucose was 5.9 mmol/l and rose to 8.6 mmol/l (insulin 960 pmol/l) two hours after a standard glucose tolerance test with 1.75 g/kg BW [12].

#### Family II

**Figure 2:**
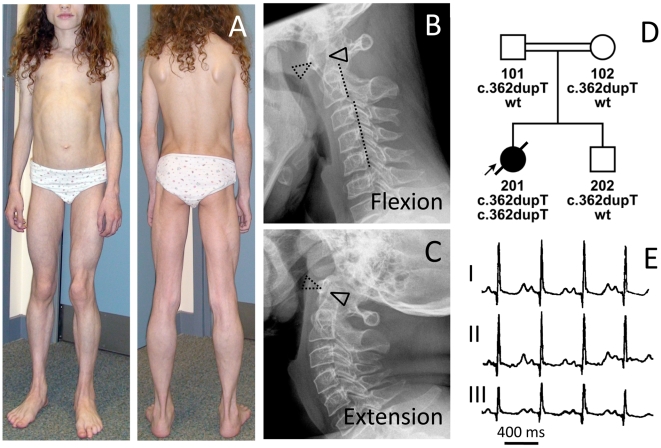
Phenotype of patient FII:201 from the UK. (A) Image of the 12-year-old patient with generalized lack of subcutaneous fat and prominent muscle hypertrophy especially of the thigh (see also Figure S1), masticatory and paraspinal muscles. The patient presented with spinal rigidity and lumbar hyperlordosis. The veins generally appeared thickened and prominent (phlebomegaly). Atlanto-axial instability of the patient during flexion (B), and extension (C) of the cervical spine. In flexion, the gap between the ***anterior arc of the atlas*** (dotted triangle) and the ***odontoid process of the axis*** (closed triangle) opens up 7 mm. The posterior arch of the axis appears dysplastic. During flexion the posterior margins of the cervical vertebrae are misaligned (dotted line). There is marked loss of bone mineral density and increase of translucency of the cervical vertebrae due to osteoporosis. (D) Pedigree of the consanguineous family and genotypes of the family members. (E) ECG of the patient with a QTc (Bazett) of 501 ms.

**Figure 3:**
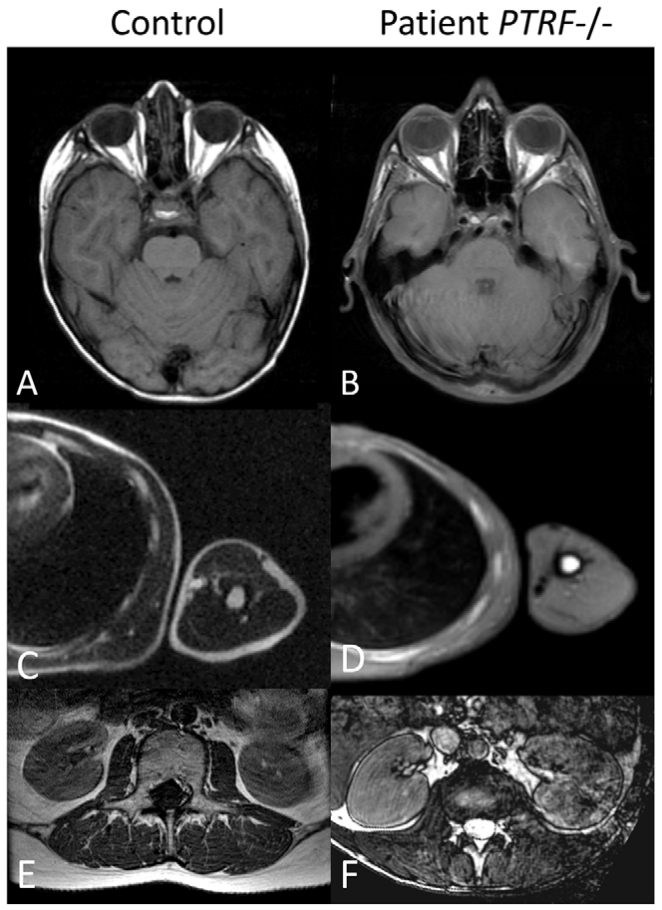
Abnormal fat distribution in patients with *PTRF-CAVIN* mutations. T_1_-weighted MR-images of patient FI:201 (B,D) and of patient FII:201 (F). On the left, the corresponding images of normal controls (A,C,E) are shown for comparison. T_1_-weighted images depict fat with high signal intensity thus giving an overview of the fat distribution. Subcutaneous fat is nearly completely lost over the peripheries, thoracic and abdominal walls and on both temporal regions. There is relative preservation of fat within the orbits. Paraspinal and perirenal fat is also reduced in bulk but relatively preserved. Fat in the bone marrow of the ribs and the *humerus* seems to be normal.

The girl (Figure 2A) was born to healthy first degree cousins (FII:201, Figure 2D) from the UK. She showed neonatal hypotonia and elevated serum creatine kinase levels (1,300 IU/l). Her appetite in early childhood was poor. At 2 years of age her weight was between the 3^rd^–10^th^ percentile with normal height. Lack of subcutaneous fat (Figure 3F) and pronounced muscle bulk led to the diagnosis of lipodystrophy. She complained of stiff legs, neck flexor weakness, dizziness and involuntary muscle movements, which were later diagnosed as rippling muscle disease. Her cognitive functions were normal. At 7 years of age she experienced periodic episodes of headache, abdominal pain, constipation and vomiting. At 10 years of age she developed proximal muscle weakness and had problems climbing stairs. Muscle stiffness, exercise-induced myalgia, PIRCs and spontaneous muscle rippling progressed over the next two years, without any evidence of myotonia. Cardiac loop recording after two syncopes revealed sinus arrhythmia, supraventricular and ventricular tachycardias and ventricular extrasystoles. The corrected QT-time was between 430–501 ms (Figure 2E). At 12 years of age, an acute ileus after appendectomy required a partial bowel resection and histology of the colon wall revealed massive smooth muscle hypertrophy. She suffered from frequent infections (tonsillitis) from which she was slow to recover. Atlanto-axial instability with subluxation was verified by X-ray (Figure 2B and 2C) as well as magnetic resonance imaging and a DEXA-scan revealed severe osteoporosis (Z-score -5.7) and a total whole body fat of 9.7%. At 12 years of age her symptoms were accompanied by hepatomegaly with elevated transaminases (ALT 128 U/l, AST 105 U/l) and signs of fatty infiltration on ultrasound, elevated C-peptide (2,569 pmol/l; N 190–990) and insulin levels (331 pmol/l) at a fasting glucose of 5.8 mmol/l. Insulin resistance was considerably increased with HOMA-IR indices ranging between 5.4 and 11.8 without evidence of *acanthosis nigricans*. A glucose tolerance test showed a fasting glucose of 5.6 mmol/l and elevated glucose (8.0 mmol/l) and insulin levels (1,241 pmol/l) two hours after glucose administration. Dyslipidemia was present with *elevated* triglycerides (3.7–8.5 mmol/l), an abnormal cholesterol profile (HDL 0.4–0.7 mmol/l, LDL 2.1–2.3 mmol/l) and reduced apolipoprotein A1 (0.53–0.81 g/l). Serum leptin levels (between <0.1 to 0.2 µg/l) were below normal. At the age of 13 years the patient collapsed while playing and died from sudden cardiac death due to ventricular fibrillation, which was still seen in the ambulance but impossible to cardiovert.

#### Further families from Oman

**Figure 4:**
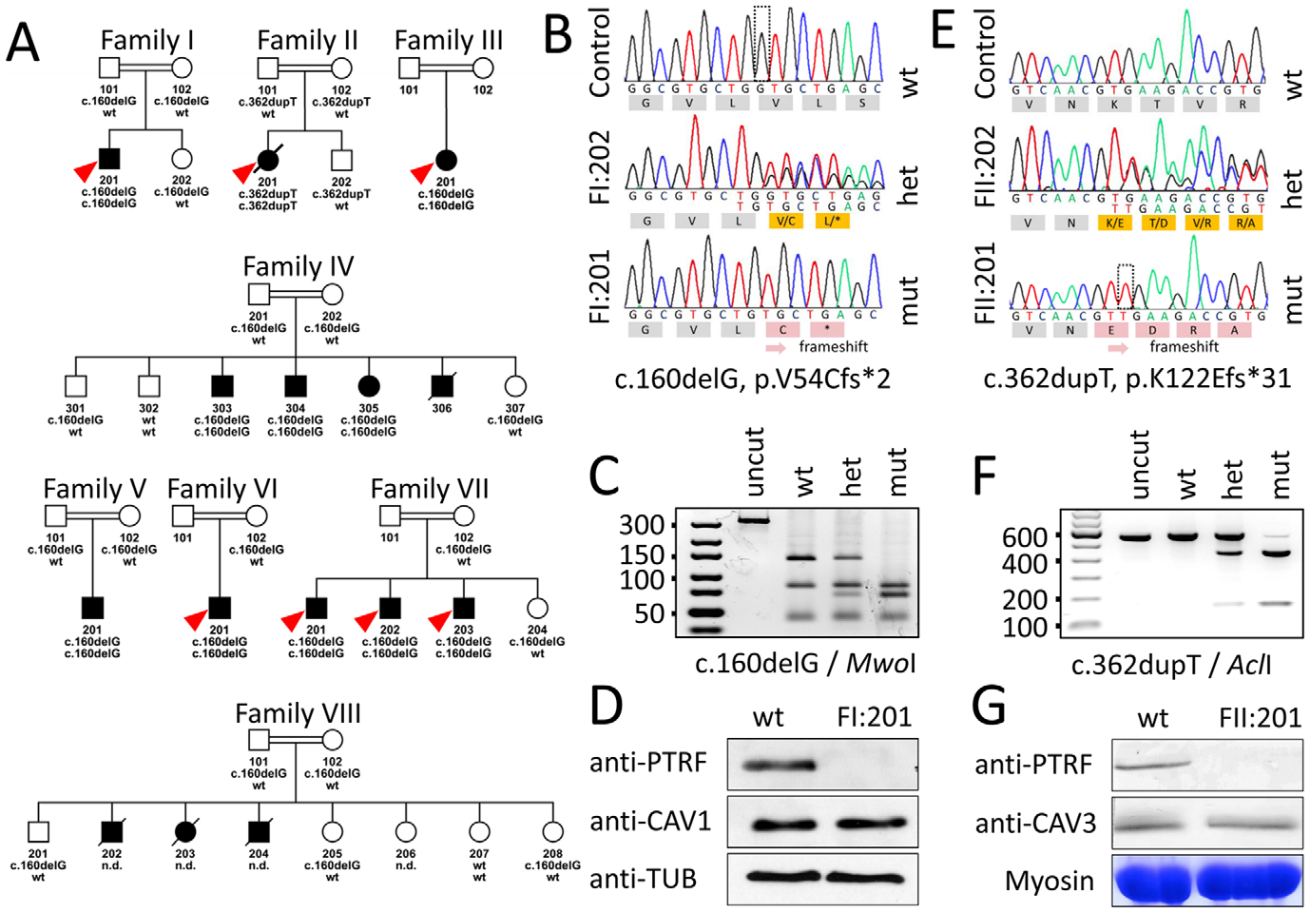
Family pedigrees and molecular genetic characterization of the *PTRF-CAVIN* mutations. (A) Pedigrees of all investigated family members. With the exception of Family II, who came from the United Kingdom, all other families originated from Oman. The red arrowheads indicate the patients included for homozygosity mapping. The genotype of each individual is marked below the symbol. (B–D) Molecular findings in Family I in whom we found a deletion of a guanine residue at nt160 (hatched box) leading to a shift in the open reading frame. (C) Verification of the mutation by restriction enzyme analysis. The mutation creates a *Mwo*I restriction site cutting the 137 bp band into 67+70 bp if the mutation is present. (D) Western blot of cultured fibroblasts from patient FI:201. PTRF-CAVIN immunoreactivity is completely absent, whereas caveolin-1 immunoreactivity is normal. The bottom panel shows the β-tubulin staining as loading control. (E–G) Molecular findings of Family II, in whom we discovered a duplication of a thymine residue at position 362 (hatched box) with subsequent frameshift. (F) Verification of the mutation by restriction analysis. In the presence of the mutation *Acl*I cleaves a 539 bp band into 415+124 bp. (G) Western blot of muscle tissue from patient FII:201. Again, PTRF-CAVIN is completely absent from muscle, whereas there is no difference in caveolin-3 immunoreactivity. The bottom panel shows the myosin band as the loading control.

We additionally investigated 9 Omani patients (pedigrees on Figure 4A), their siblings and parents that had been described previously [7]. All the patients had features of muscular hypertrophy and congenital generalized lipodystrophy without evidence of *acanthosis nigricans*. In 7 out of 9 patients muscle rippling or mounding was observed. With the exception of one patient (FV:201) all had to undergo surgery for hypertrophic pyloric stenosis. Five patients (FII:201, FIV:306, FVIII:202, 203, and 204) had abruptly died in their teens, most probably due to sudden cardiac death. As all the children lived in remote tribal areas of the Sultanate Oman, a more thorough clinical and *post-mortem* investigation of these patients was impossible. None of the heterozygous individuals (parents and siblings) showed any signs of lipodystrophy, cardiac arrhythmia or other features of the disease. A synopsis summarizing clinical and laboratory features of our patients and the patients published by Hayashi *et al.* [13] can be found on Table S1.

### Homozygous *PTRF-CAVIN* mutations are pathogenic

Data analysis of the Affymetrix GeneChip SNP data with *HomozygosityMapper* [14] delineated a single 2 Mbp region on chromosome 17 between SNPs rs9903086 and rs17531431 that was homozygous in all seven index patients (red arrowheads in Figure 4A) and could be verified with microsatellite markers. Prioritizing the 74 genes in this interval with *GeneDistiller* [15] for an expression in primarily affected tissues (adipocytes, smooth and heart muscle), which was above three times the median intensity, produced *PTRF-CAVIN* as a single hit. This gene was an attractive candidate because it had been shown to interact with caveolin-1 [16], whose mutations cause lipodystrophy [5] and with caveolin-3, whose mutations cause rippling muscle disease [17]. Additionally, a *Ptrf-Cavin* knockout-mouse exhibited low body fat, dyslipidemia, glucose intolerance, and complete absence of caveolae [18]. We thus sequenced the two coding exons, intron-exon boundaries and the entire 5′ and 3′UTR of *PTRF-CAVIN* (ENSG00000177469).

In both index patients we discovered homozygous mutations of *PTRF-CAVIN* (Polymerase I and transcript release factor/Cavin) that affected the open reading frame, led to a premature termination codon and subsequent complete absence of the protein from fibroblasts and muscle (Figure 4). Homozygosity for the c.160delG mutation was verified in all patients with lipodystrophy from further six Omani families. Homozygous mutations showed complete penetrance in all families. The presence of the mutations was verified by RFLP-analysis *via* mutation dependent restriction sites (Figure 4C and 4F) Both mutations were absent in 476 chromosomes of healthy individuals from Oman (n = 142) and from Central Europe (n = 96), thus excluding a common polymorphism.

### Absence of PTRF-CAVIN protein from patient cells and tissue

**Figure 5:**
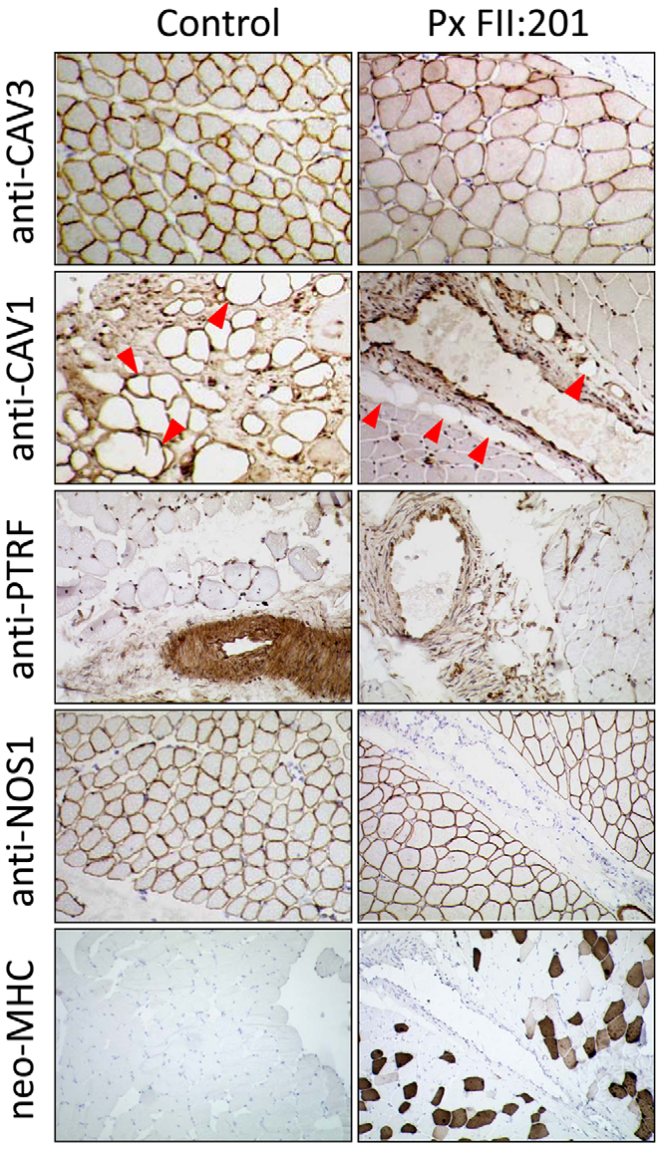
Immunohistochemistry of a muscle biopsy specimen from patient FII:201. In the patient caveolin-3 expression in skeletal muscle fibers was reduced and irregular; caveolin-1 staining of intramuscular fat cells (red arrowheads) was completely absent. In the control, strong PTRF-staining can be seen in the walls of the small arterioles, representing the smooth muscle layer. It is virtually absent in the patient, *nota bene:* The nuclear staining of the anti-PTRF-antibody is unspecific. In the patient the intensity of the subsarcolemmal anti-NOS1 staining seems to be stronger and less patchy than in the control muscle. Overall the muscle of the patient shows myopathic changes, mild variation in fiber sizes without necrosis, inflammation or fibrosis and increased regeneration. These regenerating fibers (ca. 25%) are marked through positive staining for neo-MHC, an isofom of the myosin heavy chain protein that is characteristically expressed in neonatal muscle and in regenerating fibers.

To verify the absence of PTRF-CAVIN at the protein level we performed a Western blot with protein-extracts from patient fibroblasts (FI:201) and skeletal muscle (FII:201) and probed it with anti-caveolin-1 and anti-PTRF antibodies. PTRF-CAVIN protein was completely absent from fibroblasts and skeletal muscle, whereas the caveolin-1 and caveolin-3 abundance was unchanged (Figure 4D and 4G). Immunohistochemistry of patient muscle showed absence of PTRF-CAVIN from the smooth muscle layer of the intramuscular small vessels (Figure 5). As caveolae are particularly abundant on adipocyte membranes [19], we searched for fat cells in the muscle of patient FII:201 and found complete absence of caveolin-1 immunoreactivity from the adipocyte cell membranes (red arrowheads on Figure 5). As caveolin-1 is essential for lipolysis, lipid droplet formation and lipoprotein metabolism [20] and patients with *CAV1* mutations suffer from lipodystrophy [5] we thus show that a severe reduction of caveolae *per se* may lead to a similar adipose tissue phenotype. In contrast, subsarcolemmal caveolin-3 immunoreactivity was still present, albeit at reduced levels and in a patchy distribution (Figure 5). The patient muscle showed 25% regenerating fibers as verified by reemergence of the neonatal myosin heavy chain isoform (neo-MHC).

### Severe reduction of caveolar numbers

**Figure 6:**
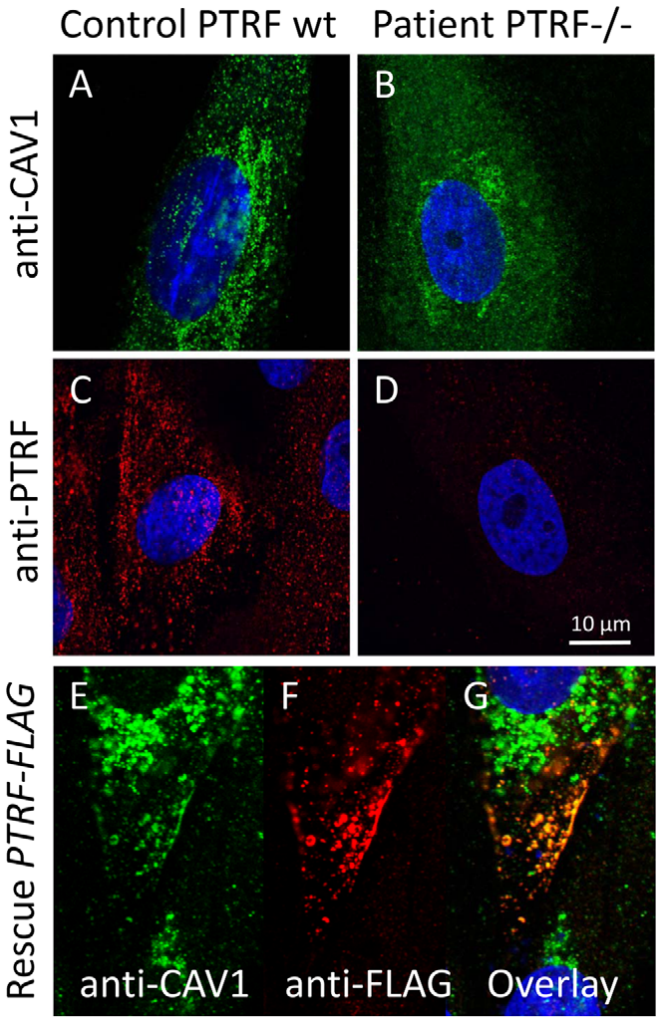
Cell-biological consequences of PTRF-CAVIN depletion. (A) Confocal microscopic image of the punctate distribution of caveolin-1 which labels the caveolae on the surface of a fibroblast. (B) Severe reduction of the punctate caveolin-1 distribution in the absence of PTRF-CAVIN and its unstructured distribution within the cytoplasm. (C) Normal punctate distribution of PTRF-CAVIN on the fibroblast surface. (D) Absence of all PTRF-CAVIN immunoreactivity on a patient fibroblast. (E–G) Each panel depicts two patient fibroblasts, one untransfected (right) and one transfected with *PTRF-FLAG* construct (left). In the untransfected cell caveolin-1 is only found in the Golgi-apparatus. Reexpression of PTRF-CAVIN in the left cell redirects the caveolin-1 staining to the caveolae in the cell periphery where the two proteins co-localize (yellow dots in panel G).

**Figure 7:**
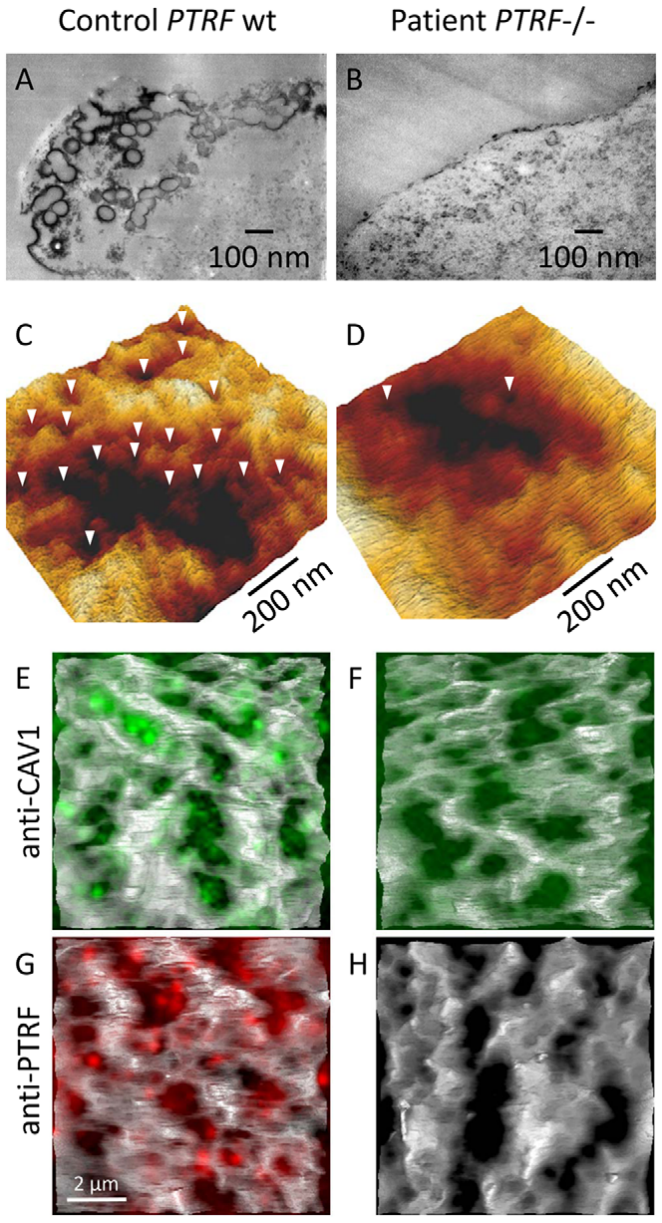
Ultrastructural analysis of caveolae on the fibroblast surface. (A,B) Transmission electron micrographs (x 37.000) of ultrathin sections from near the cell surface of a fibroblast monolayer. The cell surface is labeled with ruthenium red. In the control individual (A) numerous caveolae and indentations of the cell membrane were seen close to the cell surface. (B) In the fibroblasts of patient FI:201 only rarely coated invaginations could be found. (C,D) High resolution Atomic Force Microscopic (AFM) scans of an area of 1×1 µm on the surface of (C) control and (D) patient fibroblasts. The caveolae (white arrowheads) have a size between 50–100 nm and are predominantly located at the margins of major membrane folds. The patient fibroblasts show a smooth surface with only occasional caveolae. (E–H) Overlay of AFM-images with the respective fluorescent immunostainings from the same surface plane. On the AFM-images one can localize the caveolae by the punctate caveolin-1 staining within larger indentations of the cell membrane which disappear in the absence of PTRF-CAVIN.

We used caveolin-1 immunoreactivity as a marker for caveolae on the fibroblast surfaces. Control fibroblasts showed a punctate staining pattern at the cell periphery that dissolved in the absence of PTRF-CAVIN (Figure 6A and 6B, Figure 7E and 7F) while the perinuclear caveolin-1 staining within the Golgi-apparatus remained unchanged. As caveolin-1 was present in patient fibroblasts in normal quantities (Figure 4D) lack of PTRF-CAVIN seems to disable recruitment of caveolin-1 into the caveolar microdomains of the outer cell membrane. However, transfection of a construct into patient fibroblasts, which contained the full-length, wild-type *PTRF-CAVIN* gene cloned downstream of the eukaryotic CMV-promoter, reconstituted the punctate staining pattern at the cell periphery that corresponds to the caveolae (Figure 6E–6G). Morphometric analysis by transmission electron microscopy of the quantity of caveolae on the cell surface revealed a severe reduction by >97% in the patient's fibroblasts (Figure 7A and 7B). Complementary to this technique we employed medium and high resolution Atomic Force Microscopy (AFM) to study the cell surface of native and mildly fixated cells from patient and control. This revealed a comparatively smooth cell surface in the patient with only occasional indentations of 50–100 nm corresponding to caveolae (Figure 7C and 7D). In the patient, overlay between AFM and confocal images revealed the absence of the punctate caveolin-1 staining at the cell surface especially within the major indentations of the fibroblast cell membrane. Instead, the caveolin-3 immunoreactivity was diffusely distributed within the cytoplasm. As expected, PTRF-CAVIN immunoreactivity was entirely absent from the cell surface of patient fibroblasts (Figure 7H).

## Discussion

Here we describe eleven patients with mutations in a gene that encodes PTRF-CAVIN, which is essential for the biogenesis of caveolae. Thus the multi-facetted symptoms of our patients combine the features seen in individuals with mutations in caveolin-1 (lipodystrophy) and in caveolin-3 (myopathy). While drafting our report, we became aware of similar work by Hayashi *et al.* [13]. Using a complementary candidate gene approach they had identified *PTRF-CAVIN* mutations in five Japanese patients with lipodystrophy and myopathy with muscular hypertrophy. The cardiac and bone phenotype was not described in detail. All their patients harbored the p.K233fs mutation, four in homozygous state and a fifth patient as compound heterozygote together with a p.E176fs mutation. Those mutant proteins failed to localize to the cell membrane, however, re-emergence of caveolae after transfection of patient cells with a full-length rescue plasmid was not examined. In muscle, contrary to our results, they found a secondary reduction of caveolin-3 on Western blot. Parallel to our findings from patient fibroblasts, they also found a severe reduction of caveolae on electron microscopic images of patient muscle. This further strengthens the assumption that PTRF-CAVIN deficiency presents the phenotypic spectrum caused by generalized impairment of caveolae biogenesis.

In addition to the “classic” methods of transmission electron microscopy we here demonstrate the feasibility of Atomic Force Microscopy (AFM) in combination with immunofluorescent imaging to visualize the pathological changes brought about by an inherited defect of caveolae biogenesis. This method provides high-resolution images from the cellular surface without the need for dehydration and extensive fixation of the cells. It can even be used for live-cell imaging and may be a potential tool for real-time imaging of caveolar dynamics in the future [21].

### Multiple caveolar dysfunctions may explain patient symptoms beyond lipodystrophy

*PTRF-CAVIN* is expressed in many tissues but highest mRNA-levels are found in adipocytes, muscle (smooth, heart, and skeletal), osteoblasts, but not in neuronal tissue (BioGPS, http://biogps.gnf.org/). Hence its expression pattern is congruent with the multi-system disorder seen in our patients that spares the nervous system.

In addition to lipodystrophy, most patients showed signs of smooth muscle hypertrophy in the gastrointestinal tract leading to dysmotility, dysphagia, ileus and infantile hypertrophic pyloric stenosis (IHPS). Presently the genetic basis of IHPS is unknown, although several susceptibility loci have been discovered, with the Nitrate Oxide Synthase type 1 (*NOS1,* syn. *nNOS*) locus amongst them and a *NOS1*-knockout mouse showing a gastric outlet obstruction [22]. As NOS1 co-localizes with caveolin-1 [23] and caveolin-3 [24], we investigated the patient muscle with anti-NOS1 antibodies, and found an increased sub-sarcolemmal immunostaining (Figure 5). This finding is in accord with the data of Hayashi *et al.* who also found an overexpression of NOS1 in two muscle samples of their patients [13] as well as with results from myopathic mice with transgenic expression of mutant p.P104L caveolin-3 [25]. These findings make it unlikely that PTRF-CAVIN-deficiency acts via NOS1 depletion to cause IHPS.

Various cardiac ion channels, especially the nodal pacemaker channel HCN4 (hyperpolarization-activated cyclic nucleotide-gated potassium channel 4), the voltage-gated Na^+^-channel (Na_v_1.5, *SCN5A*), which is important for excitability and propagation of cardiac depolarization waves, and the L-type Ca^2+^-channels (*CACNA1C*) necessary for excitation-contraction coupling [26], are closely associated with caveolae and caveolin-3 (*CAV3*) immunoreactivity. Mutations of *SCN5A, CACNA1C* and *CAV3* are associated with ventricular tachycardia, long-QT syndromes (LQT3,8,9), and sudden cardiac death while mutations of *HCN4* cause sick sinus syndrome type 2 [27]. Patients FI:201 and FII:201 showed features of both arrhythmias and additionally of LQT-syndrome, which we assume may be due to the simultaneous functional dissociation of all those ion channels that are functionally dependent on caveolae. Clearly, more functional and immune electron microscopic studies of cardiomyocytes are needed to verify the effect of PTRF-CAVIN on receptor clustering and function.

The muscle weakness of our patients resembled the pattern seen in limb girdle muscular dystrophy type 1C (LGMD1C) and rippling muscle disease (RMD), which are caused by dominant-negative mutations in *CAV3* [17]. “Rippling” denotes rolling and sometimes painful muscle contractions which are not caused by sarcolemmal depolarization and thus silent on EMG [28]. One theory implicates the propagation of action potentials inside the muscle fiber through a malformed longitudinal tubular system [29]. Disruption of the T-tubular system in the absence of PTRF-CAVIN seems to be likely, because alteration of *PTRF-CAVIN* mRNA-levels considerably influenced tubular morphology [30].

Regarding the increased susceptibility of both patients to bacterial infections, it is noteworthy that caveolae have been found on stimulated B-lymphocytes (plasma cells) and that murine *Cav1* ^-/-^ B-lymphocytes showed reduced *in vitro* IgG_3_-secretion after LPS-stimulation [31]. These findings support the assumption that the structural integrity of caveolae is needed for a regular humoral immune reaction.

Finally, caveolae are abundantly present on osteoblasts and are involved in the regulation of alkaline phosphate transcription and protein activity *via* the bone morphogenetic protein-2 (BMP-2) signaling pathway [32], while caveolin-1 has a role in bone matrix calcification [33]. Disruption of both functions through absence of caveolae might be the cause for abnormal bone growth (osteopenia), reduced matrix calcification (osteoporosis) and reduced stability of the vertebrae and their ligaments (atlanto-axial instability) in our patients.

In conclusion, the diverse clinical spectrum of PTRF-CAVIN deficiency displays the consequences of a quintessential lack of functional caveolae. We have shown that the spectrum mirrors the expression pattern of the gene and the distribution of caveolar functions according to current knowledge. The presence of generalized lipodystrophy in combination with muscle rippling/mounding, intestinal obstruction and the absence of *acanthosis nigricans* should let geneticists think of PTRF-CAVIN deficiency.

Taken together, these features clearly delineate CGL4-patients from individuals with *BSCL2*, *AGPAT2*, and *CAV1* mutations [2]. Clinicians should be alerted to perform detailed cardiac investigations to search for potentially serious arrhythmias and, if positive, consider the application of an implanted cardioverter defibrillator (ICD) device.

## Methods

### Ethics statement

All patients and guardians provided written informed consent for genetic analysis according to the Declaration of Helsinki. The study was approved by the IRB of the Charité.

### Haplotype and DNA analysis

DNA was extracted from EDTA-blood, saliva or mucosal swabs with the salt extraction method [34]. Hybridization and laser scanning of GeneChip Human Mapping 250K SNP-arrays (Affymetrix) were performed according to the specifications of the manufacturer. SNP haplotpye data were analyzed for homozygous regions with *HomozygosityMapper* [14]. The candidate locus on chromosome 17 was further verified with polymorphic microsatellite markers. Prioritization of candidate genes within the homozygous region was done with *GeneDistiller* [15]. *HomozygosityMapper* and *GeneDistiller* are freely available on the Internet at http://www.homozygositymapper.org and http://www.genedistiller.org. The coding region of *PTRF-CAVIN* with its 5′ and 3′UTR was PCR-amplified with genomic primers spanning the two exons and ca. 50 bp of the intron-exon boundary on each side (Table S3). Automatic sequencing was performed with the BigDye Terminator protocol (Applied Biosystems, Darmstadt, Germany) according to standard protocols and sequences were analyzed using *MutationSurveyor* v3.10 (Softgenetics, State College, PA, USA). The mutations were further verified in the patients and excluded in normal controls by restriction fragment length polymorphism (RFLP) analysis: **c.160delG,** the primer pair 5-CCC CAC GCT CTA TAT TGT CG-3 and 5-AGC TTG CTC ACC GTA TTG CT-3 amplifies a 320 bp fragment from genomic DNA. The restriction endonuclease *Mwo*I cleaves the **wildtype allele** into ***137***+83+42+41+9+4+5 bp and the **mutant allele** into ***67+70***+83+42+41+9+4+5 bp fragments. **c.362dupT,** the primer pair 5-GTC TCC CGC TCC AGC TC-3 and 5-TGT GGG CTC ACC TGG TAG AT-3 amplifies a 540 bp fragment from genomic DNA. The restriction endonuclease *Acl*I **does not cleave** the **wildtype allele** whereas the **mutant allele** is cleaved into ***416+124*** bp.

### Western blot

Protein was extracted from patient cultured fibroblasts (patient FI:201) and muscle (patient FII:201) after homogenization in RIPA buffer with a proteinase inhibitor cocktail (Complete, Roche-Diagnostics, Basel, Switzerland), separated through denaturating SDS-PAGE with the Laemmli system and blotted on nitrocellulose membranes by the semidry method (Biometra, Göttingen, Germany). The blots were probed with anti-caveolin-1, anti-caveolin-3 and anti-PTRF as primary antibodies and corresponding peroxidase-labeled secondary antibodies. The myosin band on the Coomassie gel was used as loading control for muscle and the anti-β-tubulin band for fibroblasts. Bands were visualized by chemiluminescence. All antibodies used in this study are described in Table S2.

### Histological investigation of patient tissues and cells

Patient fibroblasts were grown to semi-confluence in DMEM in the presence of 15% FCS and penicillin/streptomycin on uncoated cover slips, washed with PBS and fixed in 4% PFA. Control fibroblasts derived from diagnostic samples for numeric chromosomal aberrations that had turned out to be normal. After permeabilization with 0,1% (v/v) Triton X-100, immunostaining was done with primary anti-caveolin-1 and anti-PTRF antibodies and appropriate secondary antibodies (Table S2) according to standard procedures [35]. Fluorescent microscopic images were recorded with a Leica SPE laser confocal imaging system (Leica Microsystems, Wetzlar, Germany).

For muscle histology tissue was flash frozen and 6 µm sections were cut and mounted onto SuperFrost Plus slides. Immunolabeling was carried out using a standard protocol. Briefly, sections were equilibrated to room temperature and washed for 15 min in PBS pH 7.3 containing 0.1% Triton X-100 for membrane permeabilization. Sections were incubated overnight at 4°C in optimally diluted primary antibodies. The diluent contained 40% FCS and 0.1 M lysine. Following 2×10 min washes in PBS/Triton sections were incubated for 90 min at room temperature in 1∶100 HRP rabbit anti-mouse immunoglobulin (Dako P260). Sections were washed, visualized with DAB and counterstained with Carazzi's haematoxylin prior to dehydration and mounting. Control muscles were supplied from patients with orthopedic surgery.

### Functional complementation of patient fibroblasts

The full open reading frame of the *PTRF-CAVIN* gene was amplified from human fibroblast cDNA with a proof-reading polymerase (PhusionTaq; New England Biolabs, Frankfurt a. M., Germany) with tailed primers (forward 5-GGT GGT GGA TCC GGT CTC CCG CTC CAG CTC-3, reverse 5-GGT GGT GTC GAC GTC GCT GTC GCT CTT GTC CA-3) which contained engineered *BamH*I and *Sal*I restriction sites (underlined). This PCR-fragment was purified by agarose electrophoresis and cloned into the *BamH*I+*Sal*I multiple cloning sites of the pCMV-Tag4a vector (Stratagene, Amsterdam, NL) thus producing a C-terminally FLAG-tagged fusion protein of PTRF. The correct cloning was verified by automatic sequencing. For transfection of adherent fibroblasts 5 µg plasmid were mixed with Lipofectamine (Invitrogen, Leek, NL) and incubated for 24 h with cells growing in the exponential phase on cover slips. Cells were then double-stained with anti-caveolin-1 (mouse-mAB) and anti-FLAG (rabbit-pAB) and images recorded as described. The proper expression of the recombinant PTRF-FLAG protein *via* the construct was verified through double labeling of the transfected mutant cells with anti-PTRF (mouse-mAB)/anti-mouse-ALEXA555 and anti-FLAG (rabbit-pAB)/anti-rabbit-ALEXA488 antibodies (Figure S2)

### Ultrastructural investigation by transmission electron microscopy

The preparation of fibroblasts for transmission electron microscopy was done as published before [16],[36]. Briefly, cells were grown on uncoated round glass cover slips in DMEM (+15% FCS) in 2 cm diameter plastic dishes to near confluence. After rinsing with PBS, cells were fixed in Na-cacodylate-buffered 2.5% glutaraldehyde (pH 7.3) containing 1 mg/ml ruthenium red (Sigma-Aldrich, Munich, Germany) for 12 h at room temperature. Ruthenium red labels the cell surface only. Other staining protocols with OsO_4_, uranylacetate or leadcitrate were omitted. The cell monolayer was embedded in Epon resin *in situ* and the cover slips were removed by flash freezing in liquid nitrogen. 70 nm sections were cut parallel to the culture substratum from the base of the cells with a microtome (Reichert Ultracut, Vienna, Austria) using a diamond knife and placed on Formvar-coated copper grids. Microscopic images were recorded with a Zeiss E905 transmission electron microscope. For quantification of the results, we counted the number of caveolae along the length of a total of 50 µm cell membrane in random samples. In control fibroblasts we found 923 and in the patient 27 caveolae per 50 µm cell membrane cumulating in a reduction of >97% in the patient.

### Ultrastructural investigation by Atomic Force Microscopy (AFM)

Atomic Force Microscopy (AFM) visualizes the surface membrane topography in living or only mildly fixated cells with a resolution in the nanometer range [37]. As opposed to scanning electron microscopy, dehydration of the samples is unnecessary, which allows simultaneous labeling with fluorescent antibodies, thus providing a powerful tool to see disease-specific changes on cell surfaces. Fibroblasts were cultured on glass bottom dishes (WillCo Wells BV, Amsterdam, Netherlands). After cells had attached and flattened, they were fixed with 4% PFA for 20 min at room temperature, followed by a 3×5 min wash in PBS. Cells were labeled with anti-caveolin-1 and anti-PTRF antibodies and subsequently with appropriate secondary fluorescent antibodies. AFM measurements were carried out with NanoWizard II (JPK Instruments, Berlin, Germany) combined with a Leica Optical Microscope DMI6000B equipped with a DFC360FX CCD camera (Leica Microsystems). The calibrated and deconvoluted fluorescent image from the same plane was then imported into the AFM software to secure the exact overlay between optical image and 3D surface structure. AFM measurements of the cell surface were performed in the tapping/intermittent contact mode with Si_3_N_4_-cantilevers of a nominal spring constant of 0.35 N/m (type DNP, JPK Instruments). Selected squares of 10×10 µm (medium resolution) and of 1×1 µm (high resolution) were scanned at 0.5 Hz. AFM images were processed using the NanoWizard II Image Processing Software v3.2 (JPK Instruments). A 3D-topography was generated and presented as a 2D-image.

## Supporting Information

Figure S1 Close-up view of the quadriceps muscles of patient FII:201. On the upper thighs the prominent reticular pattern of hypertrophied venous vessels (phlebomegaly) can be clearly seen. Enlargement of the distal diaphyses of the long bones becomes obvious through the broadening of the knees and of the finger joints, especially at the metacarpo-phalangeal joints.
(4.04 MB TIF)

Figure S2 Co-localization of the FLAG and PTRF-signals in pCMV-Tag4a-*PTRF-FLAG* transfected cells. The upper panel shows a confocal scan through the surface layer of a patient fibroblast (FI:201) that had been transfected with the *PTRF-FLAG* construct. The lower panel depicts a more detailed section at a higher magnification. The yellow co-localization in the overlay verifies the proper expression of the PTRF-CAVIN protein in the same location as the FLAG-immunoreactivity is found.
(2.72 MB TIF)

Table S1 Clinical information on the patients from this study and from Hayashi *et al.* (2009) [13].
(2.38 MB TIF)

Table S2 Antibodies used for western blot and immunolabeling.
(0.38 MB TIF)

Table S3 PCR primers used for molecular analysis of the *PTRF-CAVIN* gene.
(0.60 MB TIF)

## References

[1] Garg A (2004). Acquired and inherited lipodystrophies.. N Engl J Med.

[2] Garg A, Agarwal AK (2009). Lipodystrophies: Disorders of adipose tissue biology.. Biochim Biophys Acta.

[3] Magre J, Delepine M, Khallouf E, Gedde-Dahl T, Van Maldergem L (2001). Identification of the gene altered in Berardinelli-Seip congenital lipodystrophy on chromosome 11q13.. Nat Genet.

[4] Agarwal AK, Arioglu E, De Almeida S, Akkoc N, Taylor SI (2002). AGPAT2 is mutated in congenital generalized lipodystrophy linked to chromosome 9q34.. Nat Genet.

[5] Kim CA, Delepine M, Boutet E, El Mourabit H, Le Lay S (2008). Association of a homozygous nonsense caveolin-1 mutation with Berardinelli-Seip congenital lipodystrophy.. J Clin Endocrinol Metab.

[6] Cao H, Alston L, Ruschman J, Hegele RA (2008). Heterozygous CAV1 frameshift mutations (MIM 601047) in patients with atypical partial lipodystrophy and hypertriglyceridemia.. Lipids Health Dis.

[7] Rajab A, Heathcote K, Joshi S, Jeffery S, Patton M (2002). Heterogeneity for congenital generalized lipodystrophy in seventeen patients from Oman.. Am J Med Genet.

[8] Heathcote K, Rajab A, Magre J, Syrris P, Besti M (2002). Molecular analysis of Berardinelli-Seip congenital lipodystrophy in Oman: evidence for multiple loci.. Diabetes.

[9] Simha V, Agarwal AK, Aronin PA, Iannaccone ST, Garg A (2008). Novel subtype of congenital generalized lipodystrophy associated with muscular weakness and cervical spine instability.. Am J Med Genet A.

[10] Crotti L, Celano G, Dagradi F, Schwartz PJ (2008). Congenital long QT syndrome.. Orphanet J Rare Dis.

[11] Slaughter MH, Lohman TG, Boileau RA, Horswill CA, Stillman RJ (1988). Skinfold equations for estimation of body fatness in children and youth.. Hum Biol.

[12] Sinha R, Fisch G, Teague B, Tamborlane WV, Banyas B (2002). Prevalence of impaired glucose tolerance among children and adolescents with marked obesity.. N Engl J Med.

[13] Hayashi YK, Matsuda C, Ogawa M, Goto K, Tominaga K (2009). Human PTRF mutations cause secondary deficiency of caveolins resulting in muscular dystrophy with generalized lipodystrophy.. J Clin Invest.

[14] Seelow D, Schuelke M, Hildebrandt F, Nurnberg P (2009). HomozygosityMapper-an interactive approach to homozygosity mapping.. Nucleic Acids Res.

[15] Seelow D, Schwarz JM, Schuelke M (2008). GeneDistiller-distilling candidate genes from linkage intervals.. PLoS One.

[16] Hill MM, Bastiani M, Luetterforst R, Kirkham M, Kirkham A (2008). PTRF-Cavin, a conserved cytoplasmic protein required for caveola formation and function.. Cell.

[17] Betz RC, Schoser BG, Kasper D, Ricker K, Ramirez A (2001). Mutations in CAV3 cause mechanical hyperirritability of skeletal muscle in rippling muscle disease.. Nat Genet.

[18] Liu L, Brown D, McKee M, Lebrasseur NK, Yang D (2008). Deletion of Cavin/PTRF causes global loss of caveolae, dyslipidemia, and glucose intolerance.. Cell Metab.

[19] Aboulaich N, Vainonen JP, Stralfors P, Vener AV (2004). Vectorial proteomics reveal targeting, phosphorylation and specific fragmentation of polymerase I and transcript release factor (PTRF) at the surface of caveolae in human adipocytes.. Biochem J.

[20] Frank PG, Pavlides S, Cheung MW, Daumer K, Lisanti MP (2008). Role of caveolin-1 in the regulation of lipoprotein metabolism.. Am J Physiol Cell Physiol.

[21] Chen L, Chu W, Xu Y, Chen P, Lao F (2009). Time-series investigation of fused vesicles in microvessel endothelial cells with atomic force microscopy.. Microsc Res Tech.

[22] Huang PL, Dawson TM, Bredt DS, Snyder SH, Fishman MC (1993). Targeted disruption of the neuronal nitric oxide synthase gene.. Cell.

[23] Sato Y, Sagami I, Shimizu T (2004). Identification of caveolin-1-interacting sites in neuronal nitric-oxide synthase. Molecular mechanism for inhibition of NO formation.. J Biol Chem.

[24] Garcia-Cardena G, Martasek P, Masters BS, Skidd PM, Couet J (1997). Dissecting the interaction between nitric oxide synthase (NOS) and caveolin. Functional significance of the nos caveolin binding domain in vivo.. J Biol Chem.

[25] Sunada Y, Ohi H, Hase A, Ohi H, Hosono T (2001). Transgenic mice expressing mutant caveolin-3 show severe myopathy associated with increased nNOS activity.. Hum Mol Genet.

[26] Löhn M, Fürstenau M, Sagach V, Elger M, Schulze W (2000). Ignition of calcium sparks in arterial and cardiac muscle through caveolae.. Circ Res.

[27] Balijepalli RC, Kamp TJ (2008). Caveolae, ion channels and cardiac arrhythmias.. Prog Biophys Mol Biol.

[28] Vorgerd M, Bolz H, Patzold T, Kubisch C, Malin JP (1999). Phenotypic variability in rippling muscle disease.. Neurology.

[29] Lamb GD (2005). Rippling muscle disease may be caused by “silent” action potentials in the tubular system of skeletal muscle fibers.. Muscle Nerve.

[30] Hansen CG, Bright NA, Howard G, Nichols BJ (2009). SDPR induces membrane curvature and functions in the formation of caveolae.. Nat Cell Biol.

[31] Medina FA, Williams TM, Sotgia F, Tanowitz HB, Lisanti MP (2006). A novel role for caveolin-1 in B lymphocyte function and the development of thymus-independent immune responses.. Cell Cycle.

[32] Hartung A, Bitton-Worms K, Rechtman MM, Wenzel V, Boergermann JH (2006). Different routes of bone morphogenic protein (BMP) receptor endocytosis influence BMP signaling.. Mol Cell Biol.

[33] Sawada N, Taketani Y, Amizuka N, Ichikawa M, Ogawa C (2007). Caveolin-1 in extracellular matrix vesicles secreted from osteoblasts.. Bone.

[34] Miller SA, Dykes DD, Polesky HF (1988). A simple salting out procedure for extracting DNA from human nucleated cells.. Nucleic Acids Res.

[35] Allan VJ, Allan VK (2000). Basic immunofluorescence.. Protein localization by fluorescence mciroscopy.

[36] Parton RG, Molero JC, Floetenmeyer M, Green KM, James DE (2002). Characterization of a distinct plasma membrane macrodomain in differentiated adipocytes.. J Biol Chem.

[37] Lucius H, Friedrichson T, Kurzchalia TV, Lewin GR (2003). Identification of caveolae-like structures on the surface of intact cells using scanning force microscopy.. J Membr Biol.

