# Supplementary figures and images for: Fatal Cardiac Arrhythmia and Long-QT Syndrome in a New Form of Congenital Generalized Lipodystrophy with Muscle Rippling (CGL4) Due to PTRF-CAVIN Mutations
Source: PLoS Genet. 2010 Mar 12;6(3):e1000874. doi: 10.1371/journal.pgen.1000874 (PMC2837386; doi:10.1371/journal.pgen.1000874)

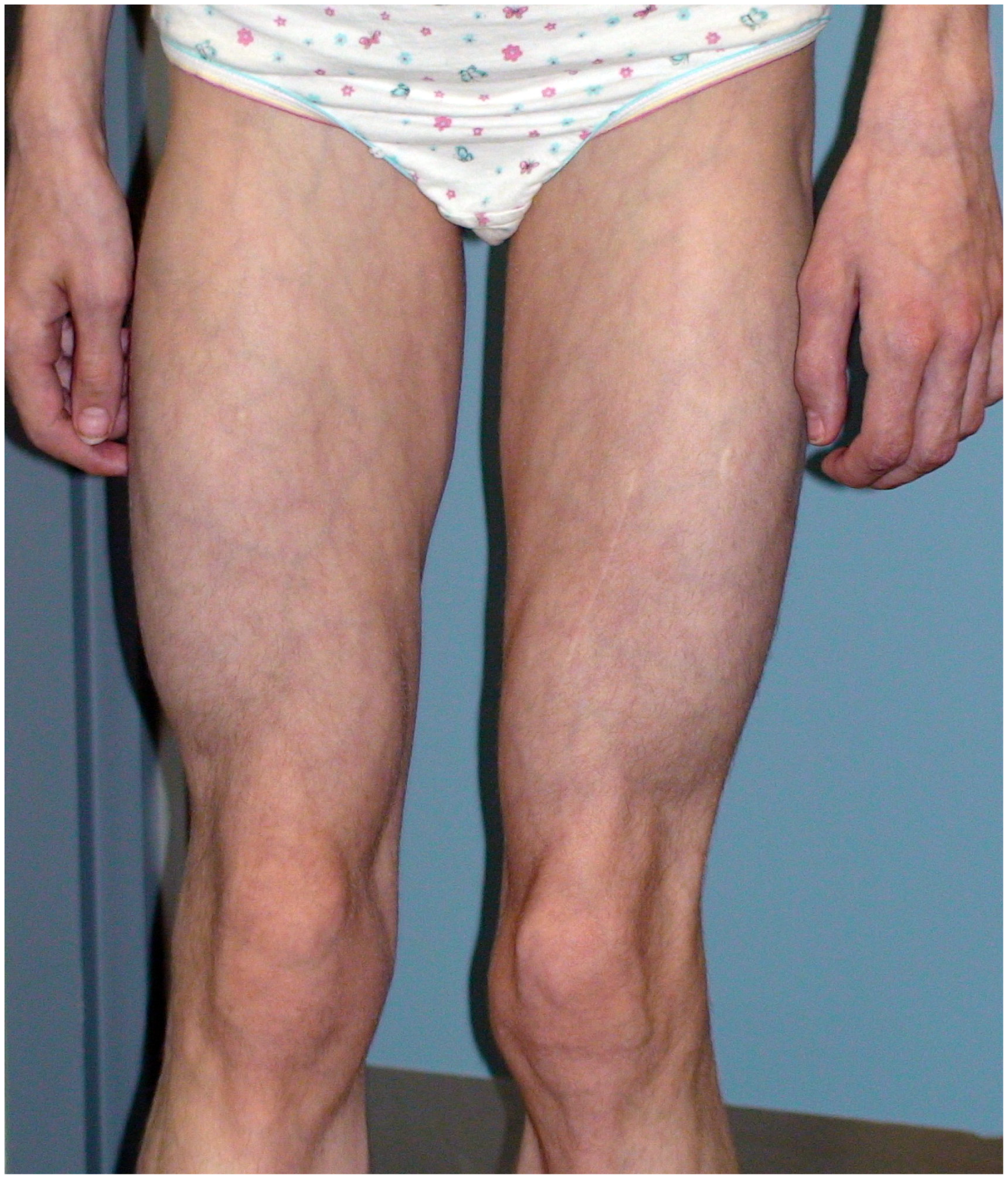

Supplement: Figure S1 — Close-up view of the quadriceps muscles of patient FII:201. On the upper thighs the prominent reticular pattern of hypertrophied venous vessels (phlebomegaly) can be clearly seen. Enlargement of the distal diaphyses of the long bones becomes obvious through the broadening of the knees and of the finger joints, especially at the metacarpo-phalangeal joints. (4.04 MB TIF) [file pgen.1000874.s001.tif]

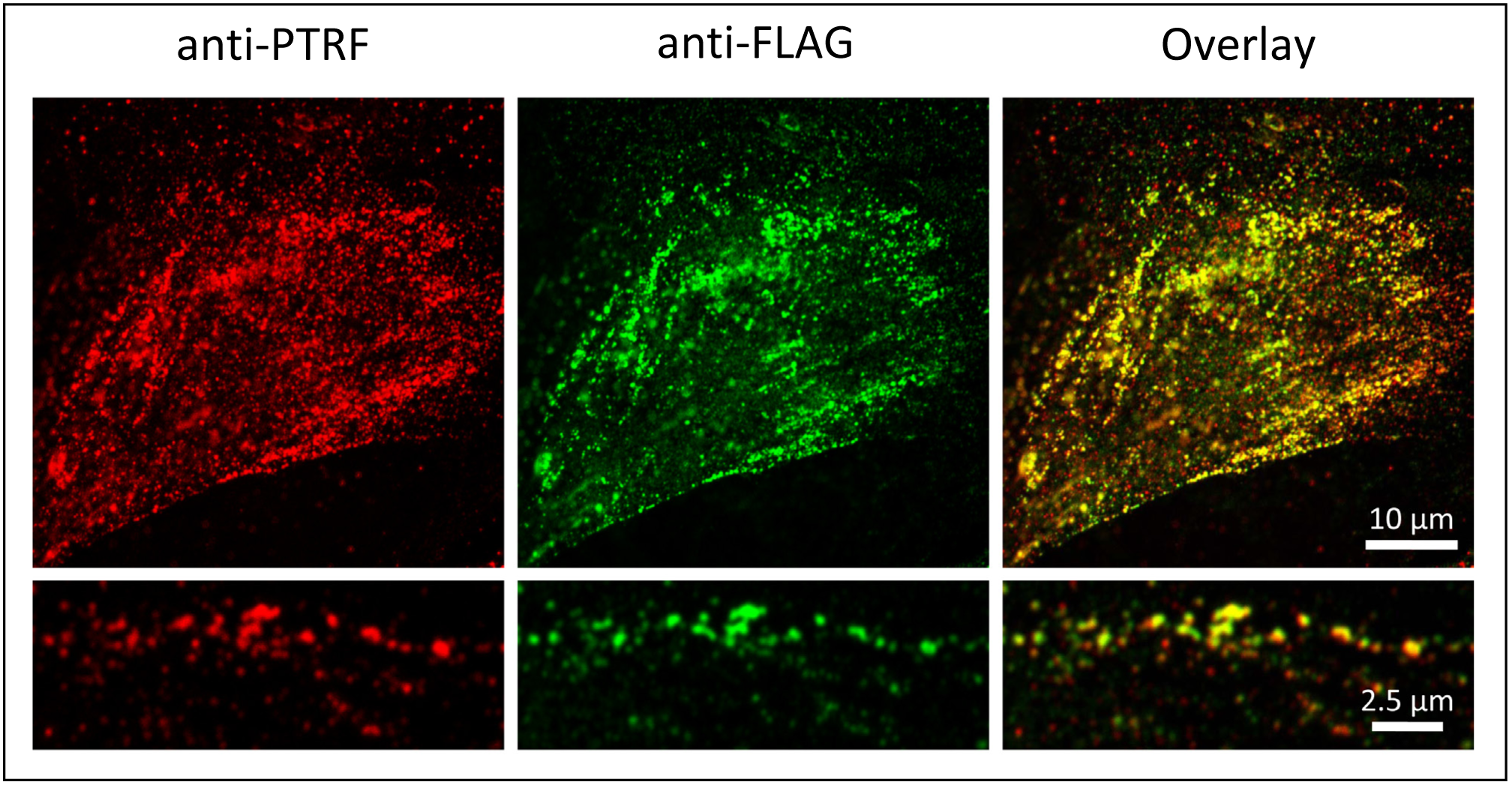

Supplement: Figure S2 — Co-localization of the FLAG and PTRF-signals in pCMV-Tag4a-PTRF-FLAG transfected cells. The upper panel shows a confocal scan through the surface layer of a patient fibroblast (FI:201) that had been transfected with the PTRF-FLAG construct. The lower panel depicts a more detailed section at a higher magnification. The yellow co-localization in the overlay verifies the proper expression of the PTRF-CAVIN protein in the same location as the FLAG-immunoreactivity is found. (2.72 MB TIF) [file pgen.1000874.s002.tif]

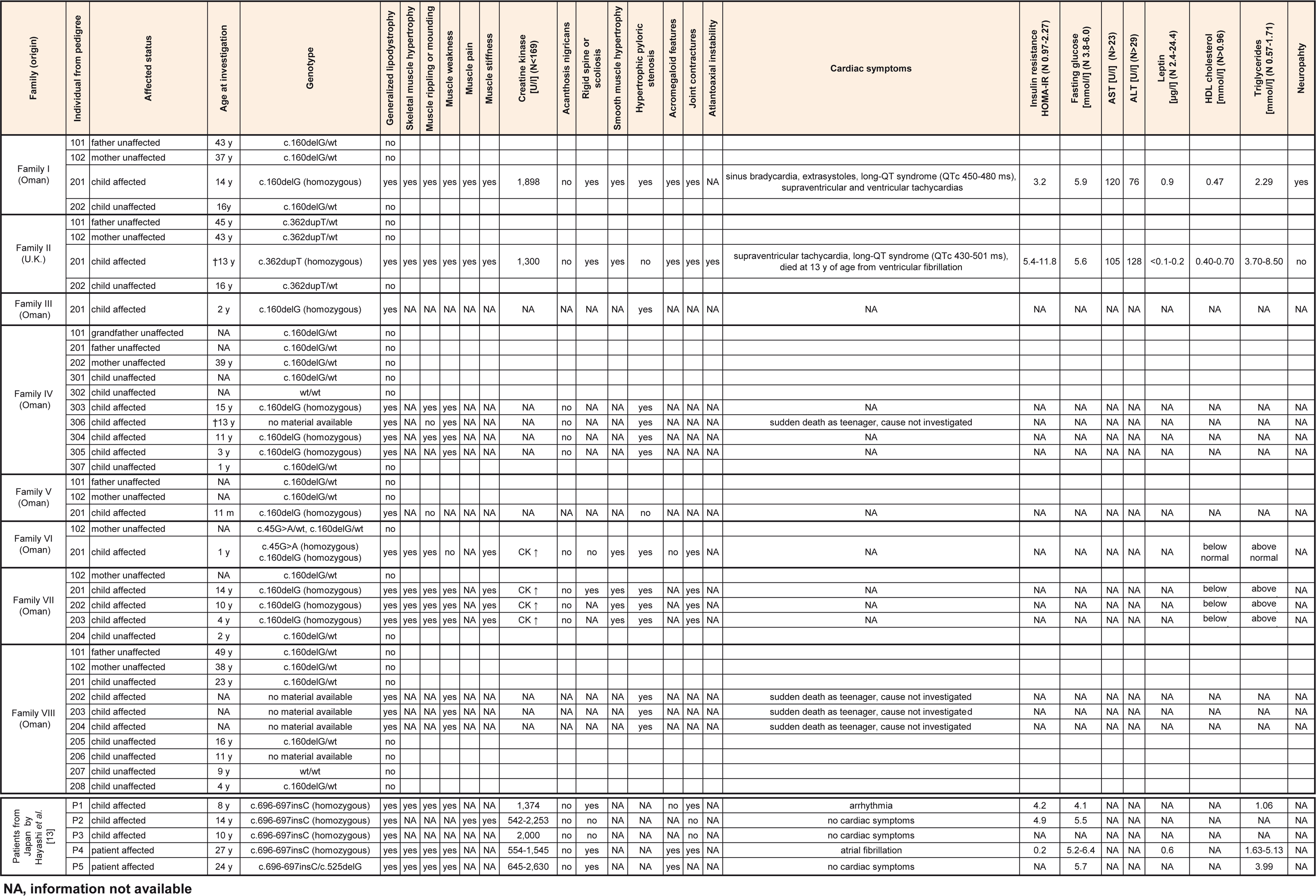

Supplement: Table S1 — Clinical information on the patients from this study and from Hayashi et al. (2009) [13]. (2.38 MB TIF) [file pgen.1000874.s003.tif]

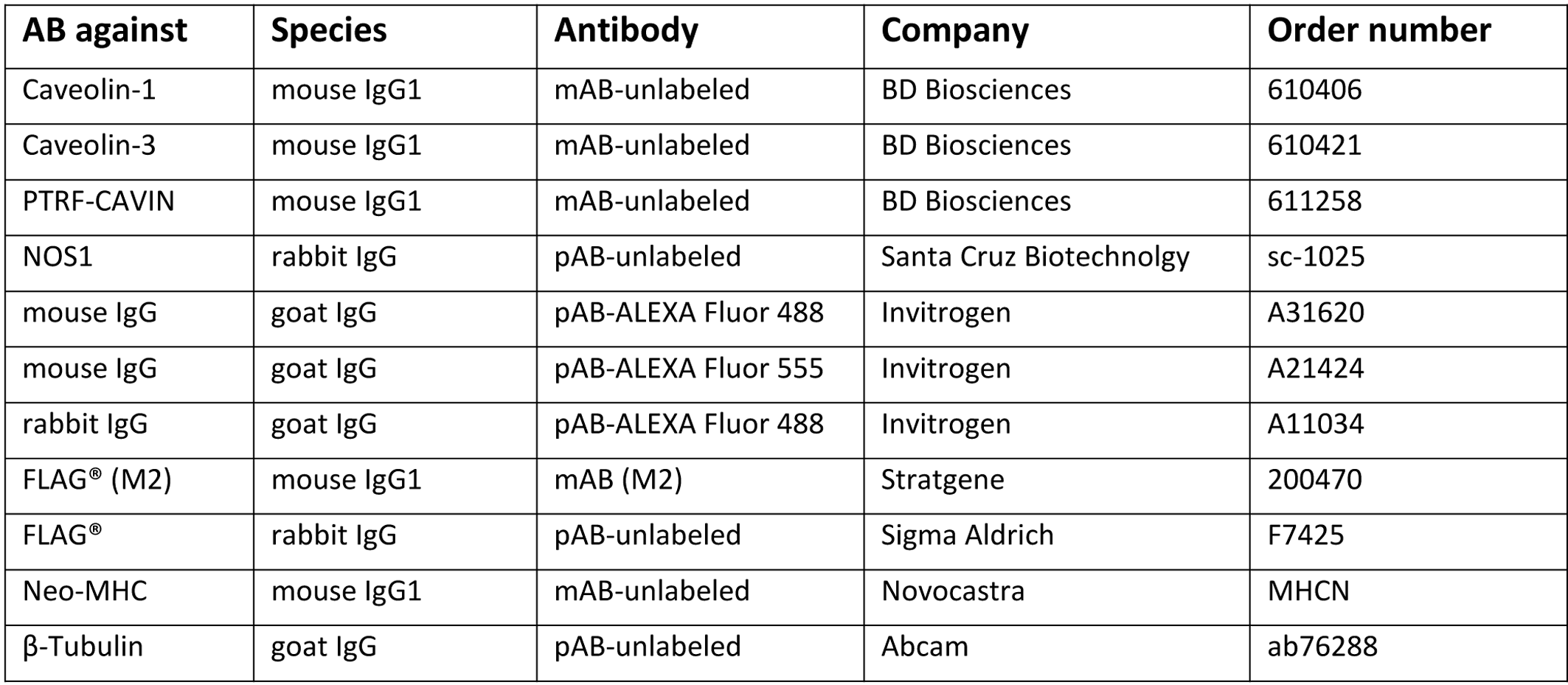

Supplement: Table S2 — Antibodies used for western blot and immunolabeling. (0.38 MB TIF) [file pgen.1000874.s004.tif]

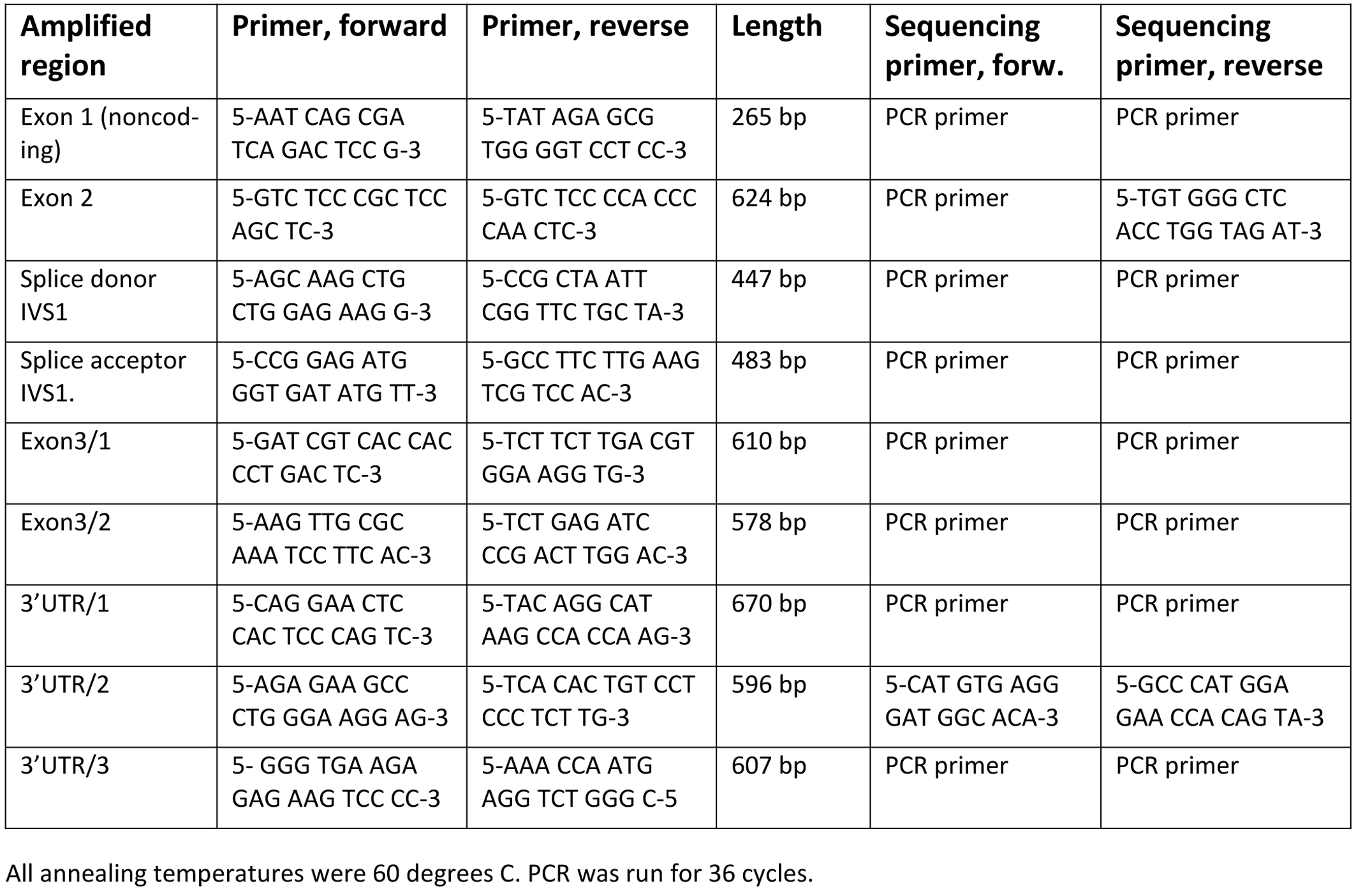

Supplement: Table S3 — PCR primers used for molecular analysis of the PTRF-CAVIN gene. (0.60 MB TIF) [file pgen.1000874.s005.tif]
